# Biofeedback electrostimulation for bionic and long-lasting neural modulation

**DOI:** 10.1038/s41467-022-33089-z

**Published:** 2022-09-09

**Authors:** Fei Jin, Tong Li, Zhidong Wei, Ruiying Xiong, Lili Qian, Juan Ma, Tao Yuan, Qi Wu, Chengteng Lai, Xiying Ma, Fuyi Wang, Ying Zhao, Fengyu Sun, Ting Wang, Zhang-Qi Feng

**Affiliations:** 1 School of Chemistry and Chemical Engineering, Nanjing University of Science and Technology, Nanjing, 210094 P. R. China; 2 Department of Orthopedic, Nanjing Jinling Hospital, Nanjing, 210002 P. R. China; 3 State Key Laboratory of Bioelectronics, Southeast University, Nanjing, 210096 P. R. China

**Keywords:** Neuroscience, Sensors and biosensors, Biomedical engineering

## Abstract

Invasive electrical stimulation (iES) is prone to cause neural stimulus-inertia owing to its excessive accumulation of exogenous charges, thereby resulting in many side effects and even failure of nerve regeneration and functional recovery. Here, a wearable neural iES system is well designed and built for bionic and long-lasting neural modulation. It can automatically yield biomimetic pulsed electrical signals under the driven of respiratory motion. These electrical signals are full of unique physiological synchronization can give biofeedback to respiratory behaviors, self-adjusting with different physiological states of the living body, and thus realizing a dynamic and biological self-matched modulation of voltage-gated calcium channels on the cell membrane. Abundant cellular and animal experimental evidence confirm an effective elimination of neural stimulus-inertia by these bioelectrical signals. An unprecedented nerve regeneration and motor functional reconstruction are achieved in long-segmental peripheral nerve defects, which is equal to the gold standard of nerve repair -- autograft. The wearable neural iES system provides an advanced platform to overcome the common neural stimulus-inertia and gives a broad avenue for personalized iES therapy of nerve injury and neurodegenerative diseases.

## Abstract (web summary)

Designing wereable neural invasive electrical stimulation system remains a challenge. Here, researchers provide an effective technology platform for the elimination of tricky neural stimulus-inertia using bionic electronic modulation, which is a significant step forward for long-lasting treatment of nervous system diseases.

## Introduction

Invasive electrical stimulation (iES), as an emerging bioelectronic medicine, has attracted great attention and offers broad application prospects for therapeutics of nervous system-related diseases (e.g., peripheral nerve injury (PNI), parkinson’s syndrome, and hysterical paralysis, etc.)^1–3^. However, conventional iES usually causes excessive charges accumulated on cell membrane, and thus to result in neural stimulus-inertia^4–7^. It will induce various complications (e.g., inflammation, immune rejection, pain, etc.) or even growth inhibition of injured nerve^8–10^. Recognizing these defects, we seek solutions and turn to nature for inspiration. Notably, endogenous electrical-neural signals will not trigger neural stimulus-inertia owing to its inherent biological self-adjusted feature, and, conversely mediate the communication of excitable cells and evoke network maturation in the neurons^11–16^. Therefore, if external electrostimulation could mimic the self-adjusted feature of endogenous nerve responses, it would respond to changing physiological need and eliminate neural stimulus-inertia, eventually enabling the long-lasting activation of the nerve regeneration.

**Fig. 1:**
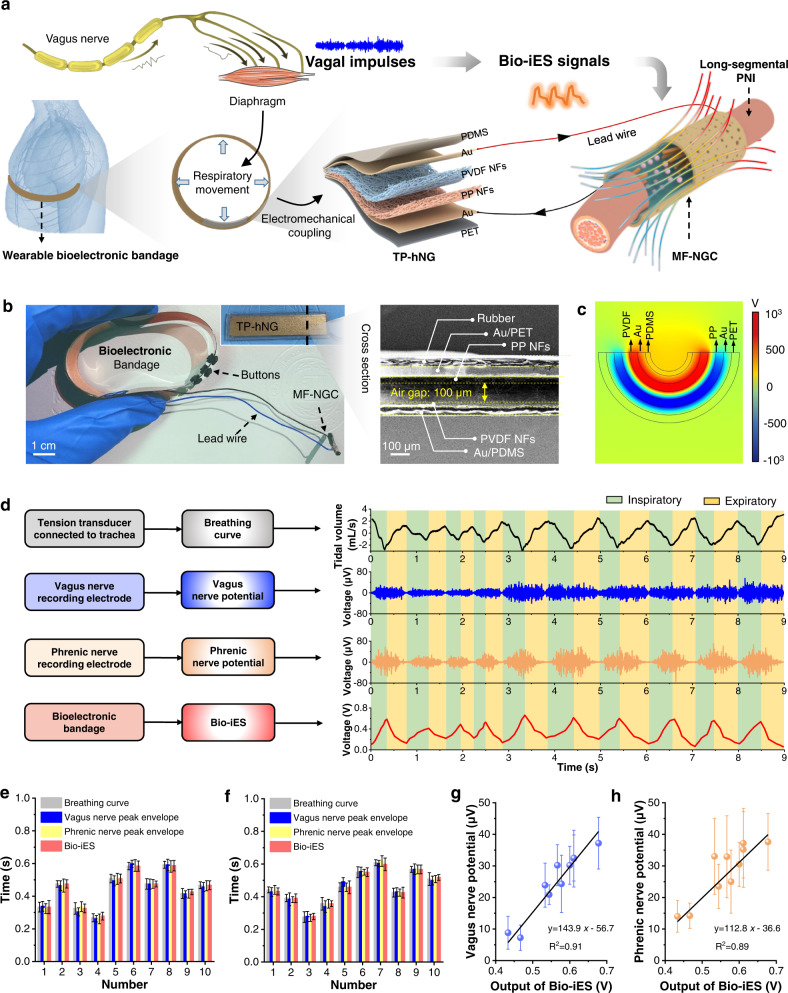
Construction of bioelectronic bandage and validation of its correlation with respiratory tract vagus nerve impulses. **a** Schematic diagram of working principle converting respiratory movement into real-time Bio-iES signals synchronized with vagus nerve impulses peak envelope, and the Bio-iES is transmitted to the defected nerve through MF-NGC. **b** Optical image of bioelectronic bandage showed miniaturization and flexibility. SEM image displayed a cross-section of bioelectronic bandages. **c** FEA analysis of potential generated in TP-hNG. **d** Synchronism measurement between Bio-iES signals (red line) and vagus (blue line) and phrenicus nerve impulses (orange line) peak envelope. Statistics of inspiratory duration **e** and exhalation duration **f** recorded from breathing curve (black line), vagus nerve peak envelope and phrenic nerve peak envelope and Bio-iES signals, respectively; **g** linear fit between Bio-iES voltage peak-to-peak and vagus nerve peak envelope potential amplitude; **h** linear fit between Vpp of Bio-iES and phrenic nerve peak envelope potential amplitude, black line indicated the linear fitting curves.

Recently, electromechanically coupling nanogenerator (NG) technology can convert transient biomechanical deformations into pulsed electrical signals^17–22^, so it is expected to correlate physiological behaviors with electrostimulation^23–28^. With this foundation, we present a neural iES system composed of triboelectric/piezoelectric hybrid NG (TP-hNG)-based elastic bioelectrical bandage and implantable polyethylene dioxythiophene (PEDOT) biodegradable multifunctional nerve guide conduit (MF-NGC). Driven by autonomic nerves system-controlled respiratory locomotion, it can yield synchronously pulsed electrical signals that directly synchronized the stimulation amplitude and frequency of respiratory behaviors, thus achieving the unique biofeedback function adapting to different physiological states (Fig. 1a). Compared with conventional square-wave iES, these bionic iES (Bio-iES) signals are able to realize dynamic modulation of opening/closing of voltage-gated calcium channels (VGCC), favoring the axonal elongation and development of nerve cells. Especially, as a demonstration of in vivo application, we further deliver these Bio-iES signals into a long-segment PNI (15 mm defect) through the MF-NGC. Evidence demonstrates that Bio-iES can eliminate the common neural stimulus-inertia and accelerate tissue regeneration and neuromotor function recovery, close to the gold standard—nerve autograft. The iES system with biofeedback function presents a practical strategy for biomimetic modulation of the growth and development of nerve cells/tissue. More broadly, this work endows unlimited possibilities for future bioelectronic therapies, where biofeedback electrostimulation pulses can replace chemical or biological drugs for electroactive cell/tissue-related diseases.

## Results and discussion

### Construction and physiological synchronization of wearable bioelectronic bandage

Electrostimulation has been known for decades to be effective in accelerating the treatment process of neurological diseases, but practical applications are still largely limited by the cumbersome and rigid electrical systems. Given this, a wearable and elastic bioelectronic bandage is constructed with a well-designed soft TP-hNG and rubber bandage (Fig. 1b) to automatically produce Bio-iES signals under the driven of vagus nerve controlled respiratory movement. Its elasticity modulus is precisely adjusted into ~7 MPa to mimic the skin’s stiffness^29^, and thus make sure the bioelectronic bandage can be tightly and comfortably affixed to targeted site to sense diaphragm deformation. Meanwhile, to adapt to the complicated biomechanics environment of living body in daily activities, the tightness of the bioelectronic bandage enables adjustment through different mini-buttons (Fig. 1b).

Furthermore, materials and structure of the TP-hNG are also optimized to improve its sensitivity and energy conversion efficiency (Supplementary Figs. 1–4). Electrospun polyvinylidene fluoride piezoelectric nanofibers (NFs) and polypropylene electret (PP) NFs are employed as the negative and positive friction layers (Fig. 1b and Supplementary Fig. 1) respectively, owing to their huge differences in the capability of gaining/losing electrons. Meanwhile, the PVDF NFs can also yield a piezoelectric field during the compression and release of TP-hNG to enhance electron transfer between two friction layers, and thus promote its electromechanical coupled efficiency (Supplementary Fig. 2). In addition, the TP-hNG presents a mechanical asymmetry structure (hard PET as outer supporting and enveloping layer, flexible PDMS as inner) to ensure sufficient contact and separation of friction layers (Supplementary Fig. 3). Based on these endeavors, the TP-hNG-based bioelectronic bandage enables rapid generation of pulsed potential under compression deformation (Fig. 1c).

To test reliability, stability, and sensitivity of the bioelectronic bandage, a series of mechanical forces simulating respiratory pressure (1–5 kPa) and frequency (0.5–3 Hz) are applied onto the TP-hNG by a precisely controlled reciprocating motion through an automatic motor (Supplementary Fig. 4)^18,30,31^. The output electrical signals remain stable at different frequencies (Supplementary Fig. 4b) and display a linear increase with pressure (coefficient of determination *R*^2^ = 0.996, slop = 3.44 V kPa^−1^) (Supplementary Fig. 4c). Meanwhile, the TP-hNG presents excellent stability (after 36,000 cycles of contact-separation processes, no fluctuations in output electrical signals) (Supplementary Fig. 4d), and possesses high sensitivity even under ultra-weak mechanical deformation caused by water drop and human breath blowing, which still yields 0.1−0.4 V pulse electrical signals (Supplementary Fig. 4h, i). These data confirm high electromechanical conversion output, superior stability, and sensitivity of the TP-hNG, which confers the bioelectronic bandage to steadily sense respiration intensity adjustment caused by subtle changes in autonomic nerve impulses, and then produce synchronously Bio-iES signals.

To demonstrate the real-time correlation between Bio-iES signals and vagal nerve impulses peak envelope, the tidal volume respiration curve, respiratory tract vagus, and phrenic nerve discharge impulses of adult Sprague-Dawley (SD) rats, and the Bio-iES signals are recorded by multi-channel physiological signal acquisition (RM6240) and digital oscilloscope (HMO3002) simultaneously (Supplementary Fig. 5). As shown in Fig. 1d, Bio-iES signals exhibit the same frequency (~1.11 Hz) as respiration curve, respiratory tract vagus, and phrenic nerve discharge signals. In each inspiration phase and exhalation phase, the duration of Bio-iES signals are precisely matched with respiratory movement time, overall vagus, and phrenic nerve peak envelope duration (Fig. 1e, f). Furthermore, by comparing the waveforms in detail, in each breathing cycle, Bio-iES signals have the same trend with vagus and phrenic nerve discharge (increasing in the inhalation phase and decreasing in the exhalation phase). Moreover, the peak-to-peak voltage (Vpp) of Bio-iES signals show a good linear correlation with the amplitude of the respiratory tract vagus nerve potential (Fig. 1g, coefficient of determination *R*^2^ = 0.91) and the phrenic nerve potential (Fig. 1h, coefficient of determination *R*^2^ = 0.89). These results reveal that Bio-iES signals are highly related to the respiratory tract vagus nerve and phrenic nerve impulses peak envelope in terms of real-time frequency and intensity, and thus reaping the intrinsic biological self-adjusted features of the autologous neural signals. This bionic feature derived from endogenous nerve responses may eliminate excessive charges accumulated on cell membrane attributed to that it can give target cells/tissues dynamic biofeedback electrostimulation.

### Elimination of neural stimulus-inertia at the cell level

**Fig. 2:**
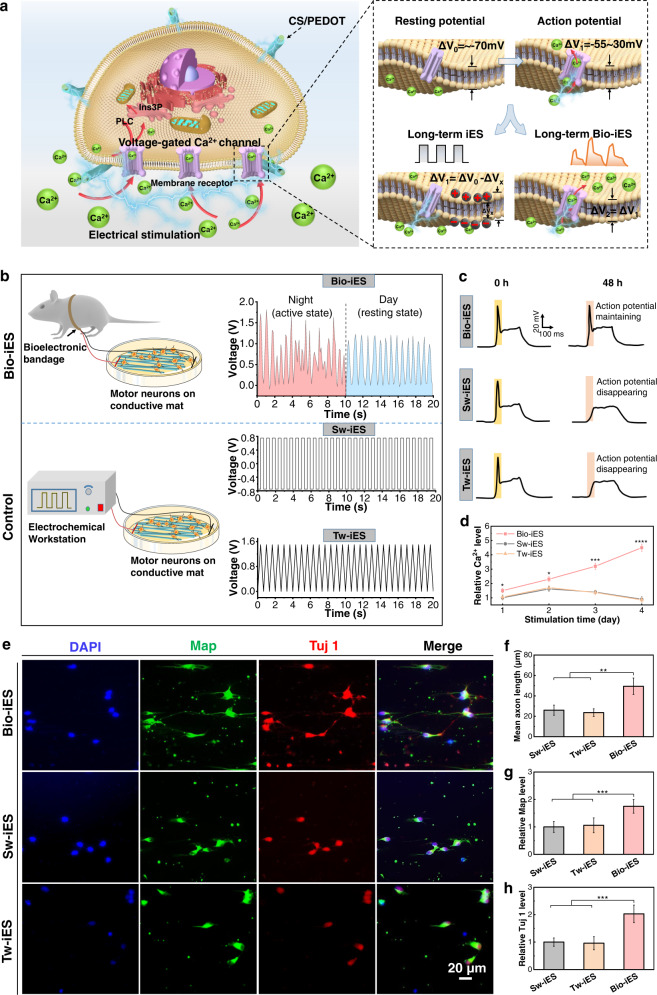
Effect of Bio-iES on cell development and maturation via eliminating neural stimulus-inertia. **a** iES induced the opening of VGCC to allow an influx of extracellular Ca^2+^ through the activation of membrane potential. Nevertheless, under the long-term electrically polarization process, commonly used constant iES signals posed the accumulation of external charges on the cell membrane, resulting in the activation of VGCC. Inversely, Bio-iES signals have the ability to retain the activation of membrane potential by dissipating the accumulation of exogenous charges. **b** Schematic diagram of Bio-iES, Sw-iES, and Tw-iES stimulating motor neurons in vitro. The waveform of Bio-iES obtained from SD rat at active (red line) and resting state (blue line), while Sw-iES and Tw-iES were provided by an electrochemical workstation. **c** Typical electrophysiological action potential response (yellow and orange areas) from the cell membranes of Bio-iES, Tw-iES and iES groups before and after electrostimulation. **d** Relative Ca^2+^ level through different stimulation times. **e** Triple fluorescence staining images of motor neurons after 2 days culture. (Red: neuronal marker protein of III β-tubulin (Tuj 1) for filopodia, green: dendrite marker protein of the microtubule-associated protein-2 (Map) for neurites, blue: neuronal nuclei). Mean axon length **f** and relative Map expression level **g** and relative Tuj 1 expression level **h** were measured from Tuj 1/Map/DAPI triple fluorescence staining. (*n* = 5, *p < 0.05, **p < 0.01, ***p < 0.001, ****p < 0.0001). All error bars indicate ±S.D.

Generally, exogenous electrical signals delivered to nerve cells/tissues, are able to locally change the cell membrane potential (resting potential: −70 mV, action potential −55−30 mV)^14,32,33^. It can trigger the opening of VGCC^4,13,14^, allow an influx of extracellular Ca^2+^ and activate calmodulin-kinases^34^, and eventually achieve targeted modulation for nerve cells and tissue^35^. However, the excessive accumulation of these exogenous charges can lead to a constant potential difference (△V_*x*_) preventing action potentials from happening, thus dampening the VGCC response, and failing the modulation of Ca^2+^ influx into cells—neural stimulus-inertia (Fig. 2a). To verify the effect of Bio-iES signals on eliminating the common neural stimulus-inertia at the cellular level, Bio-iES signals are utilized to stimulate motor neurons cultured on conductive mats in vitro (Supplementary Fig. 6). As shown in Fig. 2b, the high synchronization between Bio-iES signals and real-time respiratory movement results in a unique self-adjusting ability of the bioelectronic bandage to different physiological states. At night, the active state of rats leads to a large fluctuation at Vpp from 0.8 to 1.7 V and frequency from 1.2 to 1.8 Hz of Bio-iES signals, while rats turn into a resting state in the daytime, Vpp stabilizes at 1.25 ± 0.15 V with stable frequency (~1.5 Hz). As controls, square waves (Sw-iES groups) and Bio-iES waveform-like triangular waves (Tw-iES groups) stimulation signals are injected into motor neurons (Fig. 2b).

After 48 h of continuous electrical stimulation, motor neurons in the Bio-iES group can maintain ionic channels activity during long-term stimulation and be capable of rapidly triggering action potentials by external current pulses, whereas membrane potential differences in the Tw-iES and Sw-iES groups remain changeless and the corresponding stimulus-evoked action potential peaks disappear in current-clamp mode (Fig. 2c). Note, Bio-iES groups always exhibit higher intracellular Ca^2+^ expression levels during 4 days in culture, while an evident reduction in intracellular Ca^2+^ expression levels appears in Sw-iES and Tw-iES groups since day 2 (Fig. 2d). The phenomenon reveals a typical neural stimulus-inertia occurs under the guidance of constant Sw-iES and Tw-iES signals as the previous reports^4,10,36^. To further confirm it, the maturation of motor neurons is evaluated by using β-III-tubulin protein/microtubule-associated protein-2/2-(4-Amidinophenyl)−6-indolecarbamidine dihydrochloride (Tuj 1/Map/DAPI) triple fluorescence staining (Fig. 2e). The results show an enhanced axons development mediated by Ca^2+^ influx under the dynamic modulation of Bio-iES signals, which performs increased mean axon length (Bio-iES: 49.4 ± 8.1 μm vs Tw-iES: 23.6 ± 3.7 μm vs Sw-iES: 26.0 ± 4.9 μm) and higher neuroprotein-expression level (Fig. 2f–h). Therefore, although the waveform of the Tw-iES is similar to that of the Bio-iES, the continuous, constant-amplitude neurostimulation parameters also can induce neural stimulus-inertia, thus impeding neurite growth and maturation in motor neurons. These data clearly illustrate that the Bio-iES signals indeed eliminate neural stimulus-inertia at cell level, which results in a promoted Ca^2+^ influx and replenishment, thus accelerating neurites growth and development of nerve cells.

### Construction and application of neural iES system

Long-segmental PNI represents a significant medical problem in public health, often causes disability, and challenges surgeons^37^. Despite iES has been applied to the recovery of PNI, the motor functional reconstruction is still incomplete and often disappoints the patients due to the neural stimulus-inertia. Therefore, as an exemplary demonstration in vivo, long-segmental PNI (15 mm defect of sciatic nerve) is selected as a representative neurological diseases model to evaluate the clinical efficacy of Bio-iES in the elimination of neural stimulus-inertia for desired nerve regeneration and motor functional reconstruction.

**Fig. 3:**
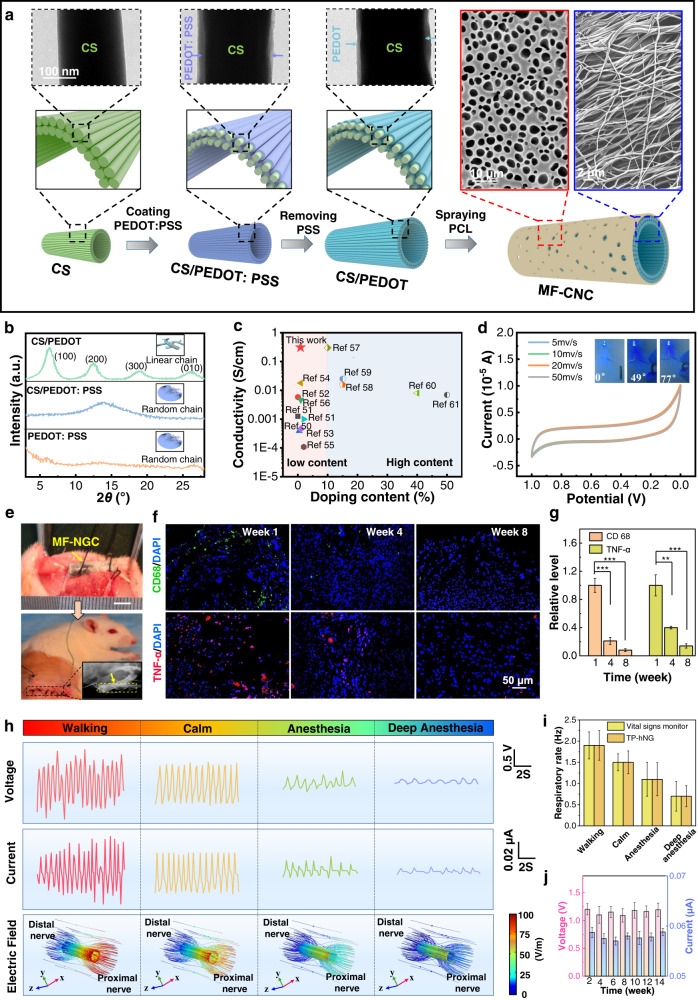
Design, physical and chemical characterization, and biocompatibility of MF-NGC. **a** Microstructure of MF-NGC. TEM image displayed a core/shell structure of CS/PEDOT NFs, and SEM images showed oriented fiber morphology of inner CS/PEDOT conducting layer and nanoporous structure of outer PCL supporting layer, respectively. **b** XRD of PEDOT: PSS membrane (orange line), CS/PEDOT: PSS (blue line), and CS/PEDOT NFs (green line). PEDOT: PSS membrane and CS/PEDOT: PSS showed amorphous structure, while the CS/PEDOT NFs displayed the obvious characteristics peaks of linear PEDOT molecular chain. **c** Compared with conventional hybrid biomaterials^51–61^, our MF-NGC reached the highest conductivity using a trace amount of PEDOT (0.8 wt%). **d** Cyclic voltammogram curve of the MF-NGC showed excellent electrochemical stability under different scan rates, the inset was photographs of MF-NGC connection with a cable and a LED bulb during bending. **e** Surgical images of MF-NGC were sutured at both ends of a 15 mm defected sciatic nerve, bar: 5 mm. Inset was an image of Small animal x-rays. **f** TNF-α (green) and CD68 (red) immunofluorescent staining of nerve tissue around MF-NGC at different time points. **g** Relative TNF-α expression level measured from CD68 immunofluorescent staining. **h** Vpp, Ipp, and electric field distribution (calculated from FEA) of output Bio-iES signals driven by respiratory motion under different physiological states of SD rats. **i** Frequency of Bio-iES signals and respiratory movement recorded by a vital signs monitor. **j** Vpp and Ipp of output Bio-iES signals recorded in 14 weeks. Data are expressed as mean values ±S.D. (*n* = 5, *p < 0.05, **p < 0.01, ***p < 0.001).

To ensure the accurate delivery of Bio-iES signals into defected nerves, an advanced MF-NGC with considerable conductivity is developed by integrating aligned core-shell chitosan/polyethylene dioxythiophene (CS/PEDOT) NFs as an inner conductive layer and porous poly(ɛ-caprolactone) (PCL) with an average pore size of 3.5 μm as an outer supporting layer (Fig. 3a and Supplementary Fig. 7-9). This can provide preferred internal environmental requirements for long-term nerve regeneration, including spatial topographical clue, porous structure, sufficient mechanical properties, and excellent biodegradability. The CS/PEDOT NFs are fabricated by a nano-interface coating of PEDOT: PSS on the surface of deprotonated electrospun CS NFs, and followed by an H_2_SO_4_ treatment process to remove insulating PSS (Supplementary Fig. 8). Thereby inducing recrystallinity of PEDOT molecular chain (Fig. 3b, enhanced peak intensity at lattice planes [100] and [010]) on the surface of CS NFs forms an ultrathin and continuous core (17 ± 4 nm, Fig. 3a). The unique core/shell structure of CS/PEDOT NFs and increased crystallinity of PEDOT result in an improved conductivity (0.042 S/cm for CS/PEDOT: PSS *vs* 0.37 S/cm for CS/PEDOT, Supplementary Table S1) and extremely stable electrochemical performance (Fig. 3c, d). These electrical features are able to ensure a stable injection of Bio-iES charges at the interface between MF-NGC and defected nerves. More importantly, compared with current conductive hybrid biomaterials, using a trace amount of doping composition (0.8 wt% of PEDOT) confers MF-NGC the highest conductivity (Fig. 3c), which also contributes to an excellent biodegeneration (Supplementary Fig. 10) and biocompatibility in vivo. At different time points after surgery (1st week, 4th week, 8th week), we assess the inflammation factors around the MF-NGC by macrophage/monocyte-specific protein (CD68) and tumor necrosis factor-α (TNF-α) immunofluorescence (Fig. 3e-g), and inflammation level returns to a normal level after postoperation 4th week (Fig. 3g). Additionally, the mechanical property of MF-NGC (elasticity modulus: 86.32 MPa, Supplementary Fig. 11) allows it to offer ideal flexibility and mechanical support during the process of nerve regeneration. All these data indicate that the MF-NGC is a superior candidate to deliver Bio-iES signals and guide the restoration of PNI.

The wearable neural iES system is built by connecting MF-NGC and bioelectronic bandage with soft Pt wires, in which the bioelectronic bandage is worn on the abdomen of experimental rats to obtain Bio-iES signals automatically (Supplementary Fig. 12). The signals at both ends of the implanted MF-NGC are recorded and analyzed as the rats are in different states including walking, calm, anesthesia, and deep anesthesia states, to confirm the physiological synchronization and self-adjusted adaptability of the neural iES system. As shown in Fig. 3h, after 3 days post-surgery, Vpp (1.5 ± 0.4 V) and Ipp (peak-to-peak current) (0.071 ± 0.02 μA) display irregular waveform with larger fluctuations in intensity at walking state, which is attributed to excessive vagal excitement and uneven breathing caused by rat motion. At calm state, the signals stay stable, no noticeable changes on Vpp (1.2 ± 0.1 V) and Ipp (0.057 ± 0.005 μA). When rats turn into anesthesia state (1% isoflurane gas), output signals perform significant reduction with irregular fluctuation due to anesthesia-induced respiratory disorders. With an increased isoflurane gas content (1.5%), SD rats enter deep anesthesia, and the Bio-iES signals present a sharp decline (blue curve) synchronizing with extremely attenuated respiratory intensity and frequency (Fig. 3i) measured by a small animal physiological recorder. These results clearly indicate that the wearable neural iES system has excellent physiological synchronization and self-adjusted adaptability, which can produce effective Bio-iES signals for nerve activation (>100 mV)^23,38,39^, regardless of any physiological states of SD rats. Furthermore, the FEA analysis (bottom in Fig. 3h) confirms that the Bio-iES signals are symmetrically distributed along the axial direction of MF-NGC, and electric flux density varied from 1.0 V/cm to 0.127 V/cm in different physiological states. Such oriented distribution can give electric cues to align the accumulation of neurotrophin to accelerate the formation of Bungner’s bands, thereby facilitating the regeneration and myelination of nerve fibers in PNI^40–42^. Subsequently, a long-term treatment (3 months) of PNI is carried out by the wearable neural iES system, which exhibits good stability throughout 3 months (Fig. 3j). During the process, Sw-iES and Tw-iES stimulation signals with constant-amplitude and frequency (1.5 V and 1.5 Hz) are provided as control groups.

### Nerve cell and tissue regeneration induced by Bio-iES signals

**Fig. 4:**
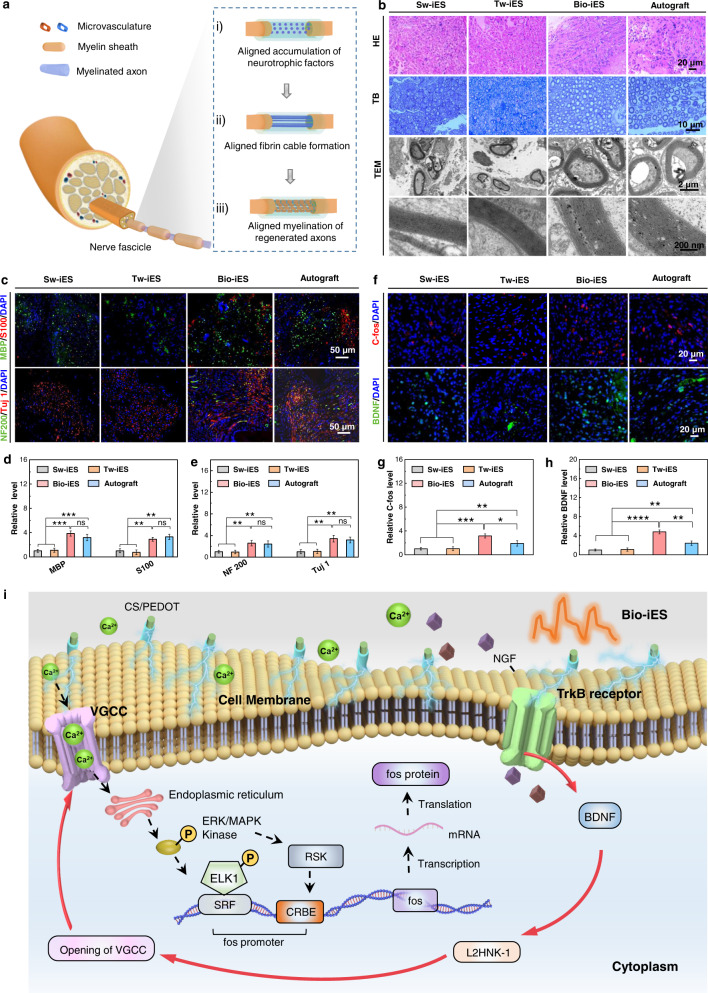
Histomorphology evaluation and nervous-specific protein expression of regenerated nerve fibers. **a** Development process of regenerated nerve: I) charged neurotrophic factors transported through the defected nerve and formed aligned accumulation along the MF-NGC; which induced II) the formation of aligned cell-free fibrin cables and further caused migration and aggregation of Schwann cells onto the cables; III) the rapidly established Schwann cells cables facilitated the myelination of regenerated neurons. **b** Hematoxylin-eosin (HE) staining (blue: Schwann cell nuclei, red: cytoplasm), toluidine blue (TB) staining, and TEM images of cross-section of regenerated nerves. **c** Triple immunofluorescent staining images of nervous tissue-specific myelin basic protein (MBP) (green), S100 (red), and nervous axon basic protein (NF200) (green) and Tuj 1 (red), nuclei (blue) in regenerated nerve fibers, bars: 50 μm. **d** Relative expression level of MBP and S100. **e** Relative expression level of NF200 and Tuj 1. **f** Immunofluorescent staining images of calcium signaling pathway C-fos (red) and BDNF (green), nuclei (blue) in regenerated nerve fibers, bars: 20 μm. Relative expression of C-fos and BDNF were shown in **g**, **h**. **i** Schematic illustration of intracellular pathway for retaining continuous activation of regeneration-associated signaling pathway under Bio-iES. Data are expressed as mean values ±S.D. (*n* = 5, *p < 0.05, **p < 0.01, ***p < 0.001, ****p < 0.0001).

Nerve regeneration is a complex and long-term process, of which accumulation of neurotrophic factors; neurite formation, and myelination of regenerated immature axons can be affected by exogenous charges (Fig. 4a). To discriminate the effects of Bio-iES, Tw-iES, and Sw-iES signals on nerve regeneration, the recruitment of Schwann cells and growth factors, and myelination are systematically evaluated. The nerve autograft by rotating 180° of severed nerve and being re-implanted into the defect area is employed as the gold standard to assess recovery satisfaction of PNI. Three months after surgery, the transected nerve in all groups are bridged by newborn nerve tissue (Supplementary Fig. 13a), but with significant stenosis in Sw-iES and Tw-iES groups (mean diameter of regenerated nerve, Sw-iES: 1.38 ± 0.15 mm, Tw-iES: 1.47 ± 0.18 mm, Bio-iES: 2.17 ± 0.21 mm, autograft: 2.22 ± 0.26 mm, Supplementary Fig. 13b). Moreover, newborn and distribution of Schwann cells and myelination of newborn axon are investigated by hematoxylin-eosin (HE), Toluidine blue (TB) staining, and transmission electron microscope (TEM). It can be seen in Fig. 4b, numerous Schwann cells (blue areas in HE staining) gathered in regenerated nerve in Bio-iES and autograft groups, but only a few amount of Schwann cells in Sw-iES and Tw-iES groups. These massive newborn Schwann cells are more conducive to the regeneration and myelination of nerves. According to the results of TB staining and TEM analysis, both Bio-iES and autograft groups have a higher axon regeneration and more myelin sheath layers in comparison to the Sw-iES and Tw-iES groups (Fig. 4b and Supplementary Fig. 14). Subsequently, the typical myelinated-specific protein (MBP and S100) and axonal-specific protein (NF200 and Tuj 1) are quantitatively assessed by triple fluorescence staining (Fig. 4c). Results present evident discrepancies between the Sw-iES and Bio-iES groups, but no statistical difference exists between Sw-iES and Tw-iES groups. The MBP and S100 expression levels in Bio-iES groups notably approach the autograft, and are 3.85 and 3.18 times of Sw-iES groups (Fig. 4d), respectively. Moreover, the regenerated nerve fibers also express higher level of typical axon protein of NF200 and Tuj 1 in the Bio-iES groups, which is 2.58 times and 3.42 times of the Sw-iES group (Fig. 4e), respectively. All these data conclusively confirm that a remarkable improvement in recruitment of the Schwann cell and myelination is achieved under the bionic dynamic modulation of Bio-iES signals contrasted to the constant Sw-iES and Tw-iES signals, and the regeneration levels of nerve cells and tissues are equal or slightly higher to the autograft. This phenomenon fully illustrates that the common neural stimulus-inertia is effectively suppressed or eliminated by the wearable neural iES system on the aspect of nerve regeneration.

To understand the biological mechanism for the elimination of neural stimulus-inertia under Bio-iES, the typical calcium-dependent signaling (e.g, markers of neuronal activity (C-fos) and brain-derived neurotrophic factor (BDNF)) is further conducted (Fig. 4f-h). Generally, nerve injuries trigger downregulation of regeneration-associated genes expression in nerve cells^39^. Exogenous electronic signals are capable of inducing the activation of the regeneration-associated signaling pathway (e.g., TrkB/BDNF pathway) to accelerate nerve regeneration. However, for commonly used constant potential/current stimulation (such as Sw-iES and Tw-iES), neural stimulus-inertia poses the inactivation of VGCC (Fig. 2a), then interrupts regeneration-associated signaling pathway, eventually causes insufficient expression of regeneration-related functional proteins and nerve growth factor (NGF)^10,43–45^. As a result, the extremely lower expression of C-fos and BDNF in Sw-iES and Tw-iES groups confirms the biological phenomenon (Fig. 4f). Notably, the BDNF and C-fos expression in Bio-iES groups are 4.34 and 2.83 times than that in Sw-iES groups after 3 months, and 1.98 and 1.66 times than that in autograft groups (Fig. 4g, h). These data fully demonstrate that Bio-iES consistently induces the influx of extracellular Ca^2+^ and successively enables the activation of the TrkB/BDNF pathway, even throughout 3 months of uninterrupted electrical stimulation. Therefore, an underlying mechanism for eliminating neural stimulus-inertia using the Bio-iES signals is probably following: under the polarization of the electric field from Bio-iES, NGF is allowed to access and be aggregated into the cytoplasm via TrkB receptor. The concentrated NGF boosts the expression of BDNF by facilitating its transcription process, leading to the expression of L2HNK-1 carbohydrates to achieve VGCC regulation. Different from procedural iES, the physiologically self-adjusted Bio-iES is able to achieve a dynamic modulation of VGCC relevant to vagus and phrenic nerve impulses peak envelope fluctuation according to real-time physiological states, thus eliminating the excessive accumulation of external charges to realize the long-lasting activation on regeneration-associated signaling pathway (Fig. 4i).

### Neural motor functional reconstruction induced by Bio-iES signals

**Fig. 5:**
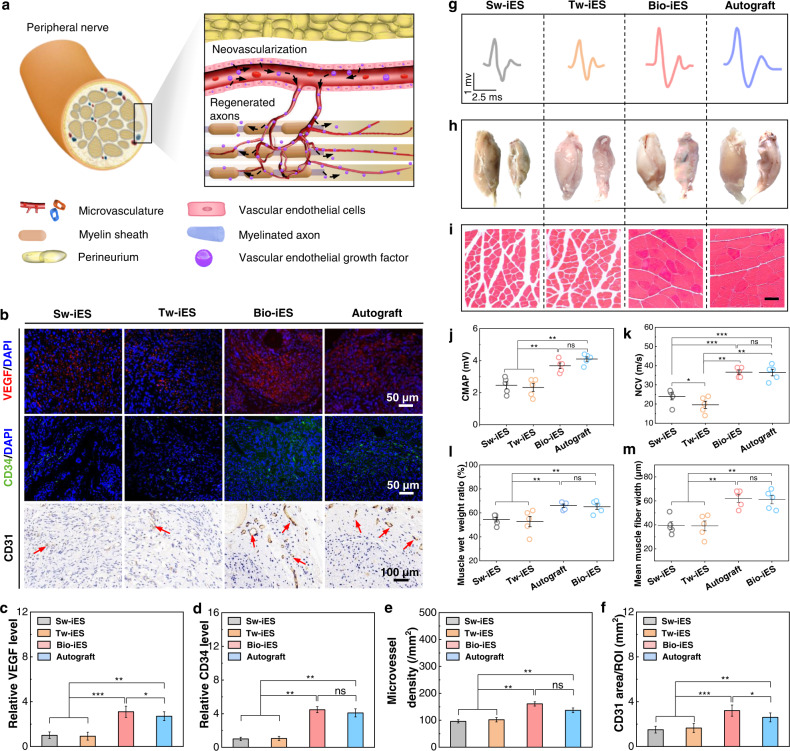
Neovascularization and neuromotor functional recovery of regenerated nerve. **a** Schematic illustration of vascular endothelial cells releasing vascular endothelial growth factor (VEGF) to induce angiogenesis and neovascularization supporting the axon regeneration of PNI. **b** Immunofluorescent staining images of vascular tissue-specific basic protein: vascular endothelial growth factor (VEGF) (green), hematopoietic transmembrane protein CD34 (red), platelet endothelial cell adhesion molecule CD31 in regenerated nerve, nuclei (blue), red arrows: CD31 cells. **c** Relative expression of VEGF. **d** Relative expression of CD34. **e** Microvessel density in the regenerated nerves was calculated from CD34 staining. **f** CD31 area in regenerated nerves calculated from CD31 staining. **g** Comparison of electrophysiological recordings of compound muscle action potential (CMAP) (gray line: Sw-iES, orange line: Tw-iES, red line: Bio-iES, blue line: autograft). **h** Photograph of collected gastrocnemius muscle. Left: normal muscle, right: pathological muscle. **i** Masson staining of the gastrocnemius muscle, red: muscle fibers; blue: collagen fibers. Bar: 100 μm. Values of CMAP and NCV are shown in **j**, **k**. **l** Statistical analysis of gastrocnemius wet-weight ratio. **m** Statistical analysis of muscle fibers width. Data are expressed as mean values ±S.D. (*n* = 5, *p < 0.05, **p < 0.01, ***p < 0.001).

It is well known that neovascularization plays a crucial role in the recovery of PNI, which can provide rich nutrition and biomolecular cycle for neural motor functional reconstruction^46–48^. The vascular endothelial cells release vascular endothelial growth factor (VEGF) to induce neovascularization, thus to support the restoration of nerve axons (Fig. 5a). Consequently, angiogenesis in newborn nerves is analyzed in detail by immunofluorescence staining of VEGF, hematopoietic transmembrane protein (CD34), and immunohistochemical of platelet endothelial cell adhesion molecule (CD31) (Fig. 5b). VEGF immunofluorescence staining shows an increased pro-angiogenic factor expression in the Bio-iES groups, which is 3.48 times of Sw-iES groups (Fig. 5c). The data of CD34 and CD31 present clear blood vessel-like structures in Bio-iES and autograft groups (Fig. 5b), and the CD34 expression in Bio-iES groups is 4.47, 4.25, and 1.1 times of Sw-iES groups, Tw-iES and autograft groups, respectively (Fig. 5d). Quantitative assessment of microvessel density from CD34 staining also shows the increased angiogenesis in Bio-iES groups (Fig. 5e). Moreover, the relatively higher CD31 area in Bio-iES groups (3.22 mm^−2^) confirms more neovascularization compared to that in Sw-iES groups (1.53 mm^−2^), Tw-iES (1.65 mm^−2^) and autograft group (2.64 mm^−2^) (Fig. 5f). It is worth noting that the enhanced neovascularization induce by the Bio-iES signals even exceeds the autograft group. This is probably because the bionic dynamic polarized electric field of the Bio-iES facilitates the recruitment of chemokine receptors CXCR4 and CXCR2 from surrounding tissue through the MF-NGC, thereby improving the migration of microvascular endothelial cells into the defect nerves^46,49^. Conversely, the CXCR4 and CXCR2 can be scarcely recognized and utilized in Sw-iES and Tw-iES groups due to neural stimulus-inertia, thus receding the neovascularization of regenerated nerve.

Furthermore, gastrocnemius recovery is quantificationally evaluated by walking trajectory analysis, compound muscle action potential (CMAP), nerve conduction velocity (NCV), gastrocnemius wet-weight ratio, Masson staining of gastrocnemius muscle (Fig. 5g–i). SD rats in Bio-iES and autograft groups exhibit better walking ability, higher sciatic function index (SFI) (−43 of Bio-iES vs −40 of autograft vs −51 of Sw-iES vs −53 of Tw-iES) (Supplementary Fig. 15). Meanwhile, Bio-iES groups also present a high level of CMAP similar to that of the autograft groups (3.7 mV for Bio-iES vs 4.1 mV for autograft vs 2.5 mV for Sw-iES vs 2.3 mV for Tw-iES) (Fig. 5g, j). Also, NCV shows the same trend (36 m/s for Bio-iES vs 39 m/s for autograft vs 24 m/s for Sw-iES vs 19.5 m/s for Tw-iES) (Fig. 5k). Moreover, gastrocnemius muscles display obvious amyotrophy in Sw-iES and Tw-iES groups (a common physiological phenomenon induced by long-term clinical constant-amplitude electrostimulation), whereas not in Bio-iES groups and autograft groups (Fig. 5h). This is also confirmed by measuring muscle wet-weight ratio (Fig. 5l) and Masson staining (Fig. 5m). In addition, abundant muscle fibers with an average diameter of 62 ± 2.7 μm appear in Bio-iES groups compared to that of 61 ± 2.3 μm in the autograft groups, 39.4 ± 3.8 μm in the Tw-iES groups and 39.6 ± 2.9 μm in the Sw-iES groups (Fig. 5m). This phenomenon of muscular hypoplasia clearly shows the occurrence of neural stimulus-inertia induced by procedural constant-amplitude electrical stimulation, which seriously inhibits the reconstruction of neuromotor function. However, the functional reconstruction induced by Bio-iES notably approaches or even exceeds the gold standard in the clinic (autograft). The significant difference reveals that the Bio-iES signals from the neural iES system are accepted facilely by nerve cell/tissue to completely eliminate the common neural stimulus-inertia, and lead to a remarkable improvement in motor function reconstruction of long-segmental PNI.

Taken together, the wearable neural iES system developed in this study provides an effective technology platform for the elimination of common neural stimulus-inertia, and thus achieving a long-lasting therapeutic effect. Such a unique feature is a significant step forward for realizing the optimum bionic electronic modulation to nervous system diseases, not only for the PNI, but also for Parkinson’s syndrome, hysterical paralysis, etc. Certainly, it should be pointed out that delivering Bio-iES signals in vivo in a body-wired electronic conduction format is just for investigating the therapeutic effect. Its clinical practicability needs further optimization because it cannot be ignored that percutaneous leads in this work are prone to patient discomfort and even other mild complications. Hopefully, for the real-world clinic application, these defects can effectively be addressed by constructing a full-implantable manner with wireless transmission technology in the future.

In summary, a wearable neural electrical stimulation system with physiological biofeedback, self-adjusted, and self-powered functions is developed to eliminate the tricky neural stimulus-inertia in clinics. Utilizing vagal impulses-controlled respiratory movement as biomechanical source, the system is capable of synchronously generating Bio-iES signals physiologically correlated with overall vagus nerve impulses peak envelopes. This unique biomimetic trait endows spontaneous and dynamic modulation of VGCC on the cell membranes in a variety of physiological states, thereby dissipating excessive charge accumulation and enabling long-lasting electrostimulation activity of regeneration-associated signaling pathways in newborn nerve cells and tissue. A near-perfect regeneration and functional reconstruction of long-segment PNI confirm the successful elimination of neural stimulus-inertia, indicating that the neural iES system offers a medium to connect autologous vagus impulse modulation with regeneration and functional reconstruction of nerve tissue first. Concurrently, this wearable form of the system provides a simple and flexible way to implement long-term nerve electrical stimulation for various kinds of neurodegenerative diseases and physical injury disease, avoiding serious surgical injury and complications caused by electronic device implantation and frequent replacement. The strategy for elimination of neural stimulus-inertia by the Bio-iES signals promotes a paradigm shift for clinical interventional electrical stimulation therapy, which will have a profound impact on intrusive bioelectronic medicine.

## Methods

### Fabrication and characterizations of electronic bandage

PVDF and PP NFs were employed as the frictional layer, while soft PDMS isolation between the triboelectric layers was used as a spacer. For more details of the fabrication, please see Supplementary Note 1. For MF-NGC, core-shell structural CS/PEDOT NFs and nanoporous PCL were fabricated by non-in situ permeability method^50^ and spray-coating method, respectively, a more detailed preparation process of MF-NGC can be found in Supplementary Note 2.

### Characterization of electronic bandage system

The output voltage and current of TP-hNG were measured by a digital oscilloscope (HMO3002) and electrochemical workstation (Chi 760e). The mechanical properties, electrical properties, stability, and degradation of MF-NGC were measured respectively. The respiration curve and vagus and phrenicus discharge impulses were measured by a multi-channel physiological signal acquisition and recording instrument (RM6240). The cell development and maturation under the electronic bandages were evaluated by continuously stimulating motor neurons. The measurement of cell membrane voltage was conducted by using a whole-cell patch-clamp technique (MultiClamp 700B). For the test method and more experimental detail please see Supplementary Notes 3–5.

### Animal experiments

The electronic bandage was affixed on the abdomen of the SD rat, while the MF-NGC was used to replace the 15 mm defect of the sciatic nerve via surgical operation. The TP-hNG and MF-NGC were connected by encapsulated soft wire to form a closed-loop system. For a more detailed process of implantation, please see Supplementary Note 6. The long-term biocompatibility of MF-NGC in vivo was assessed by CD68 and TNF-α immunofluorescence staining, respectively, please see Supplementary Note 7. The rats used in the experiments received humane care and were handled following the Institutional Animal Care and Use Committee approval protocol (JLHK 2021-0183) of the Animal Care Center at the Hospital.

### Biological assessment of regenerated nerve

The morphological observation of nerve fibers was conducted by HE, TB staining, and TEM; immunofluorescence of CD34 and VEGF, and immunohistochemical of CD31 were carried out to evaluate the neovascularization in regenerated nerve. Triple fluorescence staining of MBP/S100 and NF200/Tuj 1 was employed to evaluate the regenerated myelin sheath and myelinated axon. Immunofluorescent staining images of C-fos and BDNF were utilized to evaluate the calcium signaling pathway of basic protein. For a more detailed process of the experiment, please see Supplementary Note 8. The walking trajectory analysis, gastrocnemius wet-weight ratio, histological assessment of gastrocnemius, and electrophysiological analysis were used to measure motor functional recovery. For a more detailed process of the experiment, please see Supplementary Note 9.

### Statistical analysis

We conducted image analysis of HE staining, immunohistochemistry and immunofluorescence staining, and TEM image using Image J (NIH, USA) and Prism 8 (GraphPad, USA). All measurements except special illustration were carried out five times and all data were expressed as means ± standard deviation. Data analyses were carried out by the one-way analysis of variance followed by Tukey’s post hoc analysis with IBM SPSS 24 software. *P* value <0.05 was considered a significant level.

## Supplementary information

Supplementary Information

## Data Availability

The data supporting the findings of this study are reported in the main text or the Supplementary Information. Raw data can be obtained from the corresponding authors upon reasonable request.
