# Supplementary material for: Biofeedback electrostimulation for bionic and long-lasting neural modulation
Source: Nat Commun. 2022 Sep 9;13:5302. doi: 10.1038/s41467-022-33089-z (PMC9463164; doi:10.1038/s41467-022-33089-z)
Supplement: Supplementary file 1 — Supplementary Information [file 41467_2022_33089_MOESM1_ESM.pdf]

Supplementary Information

Biofeedback Electrostimulation for Bionic and Long-lasting Neural Modulation

Fei Jin[1, †], Tong Li[1, †], Zhidong Wei[1, †], Ruiying Xiong[1, †], Lili Qian[1], Juan Ma[1], Tao Yuan[2], Qi Wu[2], Chengteng Lai[2], Xiying Ma[1], Fuyi Wang[1], Ying Zhao[1], Fengyu Sun[1], Ting Wang[3,*] and Zhang-Qi Feng[1,*]

[1] School of Chemistry and Chemical Engineering, Nanjing University of Science and Technology, Nanjing 210094, P.R. China
[2] Department of Orthopedic, Nanjing Jinling Hospital, Nanjing 210002, P.R. China
[3] State Key Laboratory of Bioelectronics, Southeast University, Nanjing 210096, P.R. China
[†] These authors contributed equally: Fei Jin, Tong Li, Zhidong Wei, Ruiying Xiong
* Corresponding author. E-mail: fengzhangqi1981@163.com and echo2165@163.com

Supplementary Notes

Note 1. The manufacturing process of bioelectronic bandages.

PVDF nanofiber (NFs) was prepared by electrospinning the PVDF solution (MW = 5.34 kDa, Macklin Biochemical Co., Ltd.) using the #21 nozzle. The electrospinning was conducted using a high DC voltage of 18 kV with a solution flow speed of 1 mL/hour at room temperature and 35% humidity. As-received NFs were stored in a vacuum for further operation. PP electret NFs were purchased from Jinan Xingtai Environmental Protection Technology Co., Ltd. The PVDF and PP NFs were deposited with Au to form the electrode layers *via* magnetron sputtering, which then bonded to a thin PDMS and PET film, respectively. The overall size of TP-hNG was 10 mm×50 mm and a soft PDMS isolation (thickness: ~100 μm) between the contacted layers was employed as a spacer. Finally, the entire device was encapsulated by an ultra-thin PDMS. The Pt soft wire was fixed on the back of the two electrodes by applying the silver paste. The thickness of fully encapsulated TP-hNG was ~260 μm. The TP-hNG was affixed on an elastic rubber bandage to construct a flexible portable wearable electronic bandage (length：20 cm, width: 1.2 cm).

Note 2. The fabrication of MF-NGC.

The manufacturing process of aligned CS NFs conduit. CS NFs conduit was fabricated by electrospinning the mixed solution of CS (DAC 85%, MW = 100 kDa, China Hyderabad Marine Biological Engineering Co., Ltd.) and Polyethylene oxide (PEO, MW = 60 kDa, Shanghai Hairuier Chemical Technology Co., Ltd.) (mass ratio, CS/PEO=90/10). The electrospinning process was conducted using a high DC voltage of 16 kV with a solution flow speed at 0.3 ml/hour at room temperature and 25% humidity. The collector to obtain aligned CS NFs conduit was previously reported.[1] The obtained CS conduit was immersed in a saturated sodium ethoxide solution at room temperature for 15 minutes to achieve deprotonation.

The manufacturing process of CS/PEDOT: PSS conduit. The CS conduit was then coated with PEDOT: PSS solution (1.0 wt %) *via* the non-in situ permeability method to form aligned core-shell structured NFs.

The manufacturing process of CS/PEDOT conduit. The CS /PEDOT: PSS nanofibrous conduit was immersed in 0.6 mol/L concentration of H_2SO_4 (> 95% Shanghai Lingfeng Chemical Reagent Co., Ltd.) at 80 ℃ for 3-10 minutes to remove PSS.

The manufacturing process of CS/PEDOT/PCL conduit (MF-NGC). The PCL solution (10wt% in dichloromethane) was sprayed on the surface of the CS/PEDOT nanofibrous conduit using the microsyringe pump to obtain the resulting MF-NGC.

Note 3. Characterizations of MF-NGC.

The tensile tester (UTM2303) was employed to measure the mechanical properties of MF-NGC. The conductivity of MF-NGC was measured by the four-probe method *via* M3 four-probe tester (Suzhou Jingge Electronics Co., Ltd.) and then calculated according to the following formula[2]:

$$\sigma=\frac{1}{Ra\times W\times D(\frac{d}{s})} \tag{1}$$

Where W is the thickness of the sample, S represents the probe distance and D(d/s) represents the correction value, which is obtained by looking up the table based on the length of the long side and the short side of the sample.

The CV curve of the MF-NGC was measured by an electrochemical workstation (Chi 760e) and a three-electrode system. The MF-NGC were immersed in an SBF solution at 37 ℃ to simulate the degradation behavior in vivo and the mass loss of MF-NGC under dry condition were measured by a high-precision balance (BSA224S, Sartorius Scientific Instruments Co., Ltd.).

Note 4. The synchronism measurement between Bio-iES and respiratory movement

SD rats were anesthetized by the intake of isoflurane gas and then fixed on the operating table. The middle neck of SD rats was cut to expose the trachea, and a breathing tube was intubated into the trachea. The breathing tube was connected to a

tension transducer to record the tidal volume breathing curve. Meanwhile, the phrenic nerve and vagus nerve were separated to be contacted with hook-working electrodes. The grounding electrode was connected to the rats' skin. All signals were input to the multi-channel physiological signal acquisition and recording instrument (RM6240). The electronic bandage was attached to the abdomen of rats and the waveform of Bio-iES was displayed by a digital oscilloscope (HMO3002). 20 minutes after surgery, the breathing curve, phrenic and vagus nerve discharge curve, and Bio-iES signals were recorded, simultaneously.

Note 5. The growth and development on nerve cells under Bio-iES

Isolation and culture of spinal motor neurons. The spinal cords of SD rats were dissociated by adding 0.05% trypsin/EDTA for 15 minutes at 37 ℃, then, treated by trituration with a glass pipette. 10% Optiprep in Leibovitz 15 (L-15) media (Invitrogen, Long Island, NY., USA) by centrifugation to isolate motor neurons at 3400 rpm/min for 15 minutes; From the top layer after centrifugation, we collected the motor neurons. Motor neurons were washed in L-15 medium, and live cells were counted by trypan blue exclusion. Media consisted of Neurobasal (Invitrogen, Carlsbad, CA., USA) that composed with 2% B27 and the following additives: 15 μg/ml galactose, 2.5 μg/ml superoxide dismutase, 2.5 μg/ml catalase, 2.5 mg/ml albumin, 16 μg/ml putrescine, 3 ng/ml estradiol, 1X penicillin/streptomycin/neomycin, 4 ng/ml hydrocortisone, 0.01 mg/ml transferrin, 6.3 ng/ml progesterone.

Electrical stimulation of motor neurons applied by Bio-iES. Primarily, motor neurons were seeded on a cell culture plate (9×10^5 cells/cm^2) with a conductive chitosan/polyethylene dioxythiophene (CS/PEDOT) fibrous mat. The CS/PEDOT fibrous mat was fabricated successively by electrospinning and nanointerface coating techniques. The detailed parameter settings of electrospinning and nanointerface coating technique were the same as the fabrication process of multifunctional nerve guide conduit (MF-NGC) (see Supplementary text S2), but using a rotating drum as the receiver (diameter: 12 cm, rotating speed: 3000 r/min). The DMEM culture medium

(10% FBS, 1% penicillin, 1% glutamine, and 0.01% fungizone) was employed to culture cells. The culture dish was incubated in a 5% CO_2 incubator at 37 ℃ for 3 h to allow cellular adhesion. Subsequently, 4 mL of fresh medium was added to each well for further incubation. To apply Bio-iES, the cell culture dish (diameter: 55 mm) and the bioelectronic bandage (length： 20 cm, width: 1.2 cm) were connected carefully by fixed long leads (Pt wire, diameter: 0.2 mm) to construct a close-loop electrostimulation system. The bioelectronic bandage was worn on the abdomen of experimental rats and then secured with breathable clothing. Rats (6-7 weeks, 150-200g) were housed in cages with free access to water and food by using an automatic feeder. The external long leads between the culture dish and the bioelectronic bandage were wrapped around a turnable plastic reel (diameter: 70 mm) to allow rats to move freely in daily life. Two leads inside of the cell culture dish plate were bonded on the both side of the fibrous mat by conductive gel (commercial availability, purchased from Suzhou Letai Medical Technology Co, Ltd.) and then packaged by PDMS film. The PDMS film was prepared by curing the mixed solution of stock solution and curing agent (mass ratio, 10:1). Leads outside of the plate were fixed on the inside of the incubator by sterilized biological tapes (commercial availability, purchased from Suzhou Letai Medical Technology Co, Ltd.).

Meanwhile, the electrochemical workstation provided the square-wave (Sw-iES group) and triangular-wave (Tw-iES group) electrical stimulation as control groups (1.5V, 1.5Hz). Tuj1/MBP/DAPI triple fluorescence staining was used to quantitatively analyze relative neuronin expression level after electrical stimulation, and the neurite length and relative specific protein levels were measured by Image J analysis software.

Measurement of cell membrane potential. Motor neuron cultured on conductive mat were continuously stimulated by Bio-iES, Tw-iES and Sw-iES for 48h, respectively. Then, the membrane potential was measured using a whole-cell patch-clamp technique. Before measurement, the cells were washed three times and then bathed in extracellular recording solution. Electrophysiology data were recorded by MultiClamp 700B (Axon Instruments) system. The evoked membrane voltage response was stimulated by injecting current in current-clamp mode (200 pA, 270 ms).

Note 6. Implantation of MF-NGC in vivo.

Sixty SD rats (male, 150-200g, 6-7 weeks) were randomly selected for in vivo experiments. They were evenly divided into fout groups: MF-NGC with square wave electrical stimulation named as Sw-iES group; MF-NGC with triangular-wave electrical stimulation named as Tw-iES group; MF-NGC with electronic bandage named as Bio-iES group. The autograft group was set as a control group by rotating 180 °of severed nerve and being re-implanted into the defect area. For all four groups, rats were anesthetized by the intake of isoflurane gas (0.8-1.5%) and maintained with 1.0% isoflurane. Simultaneously, a 15 mm defect of the sciatic nerve was served as a nerve injury model.

Note 7. Long-term biocompatibility of MF-NGC in vivo.

The long-term biocompatibility of MF-NGC was assessed by CD68 and TNF-α immunofluorescent staining, respectively. After postoperation within two months, the sciatic nerve samples were taken out to assess the inflammatory caused by the implantation of MF-NGC. All the samples (1st week, 4th week, 8th week) were dehydrated, then embedded in paraffin, sliced into slices by using a microtome. The CD68 and TNF-αimmunofluorescence were employed to assess the inflammation level in the sciatic nerve. The observed image was obtained *via* an immunofluorescence microscope (Leica).

Note 8. Histological assessment of the regenerated nerve.

Morphology assessment of the regenerated nerve. The regenerated nerve was dissected immediately after the electrophysiological evaluation. HE staining, TB staining, and TEM analysis were used to observe the cross-sectional morphology of regenerated nerves, respectively. For HE staining, all nerve samples were fixed with 4% paraformaldehyde, then embedded in paraffin, cut into sections by using a microtome. For TB staining, and TEM, all nerve samples were fixed with 2.5% glutaraldehyde, then embedded in Epon812 resin, cut into ultrathin sections. The HE staining and TB

staining were observed by using an immunofluorescence microscope (Leica). The superfine microstructure of regenerated myelin sheath was observed *via* a TEM (China Titan) at a voltage of 80 kV. The diameter of the myelin sheath, the diameter of the myelin axon, the thickness of the myelin sheath were measured by Image J software.

Histological assessment of neovascularization on the regenerated nerve. At 12 weeks postoperatively, the regenerated sections of sciatic nerves were used for CD34 and VEGF immunofluorescence and CD31immunohistochemical assays as described above. The primary antibodies (Abcam, USA) included, anti-CD34 (1:400) and anti-CD31 (1:150), anti-VEGF (1:500 Abcam, USA). The microvessel density (MVD) and CD 31 area were measured by Image J software.

Histological assessment of typical nervous-specific protein on the regenerated nerve. The myelinated-specific protein (MBP/S100) and axonal specific protein (NF200/Tuj 1) triple immunofluorescence staining were used to assess nerve myelin protein and nerve axon protein of the regenerated nerve, respectively. The nerve segments were then fixed with 1% tetraoxide, dehydrated, and embedded in Epon812 resin. Cut the cross-section to a thickness of 4 mm (Leica EM UC 6 ultra-thin microtome) and mount on gelatin pre-coated slides. The primary antibodies (Abcam, USA) included, anti-Tuj1 (1:400), anti-NF200 (1:300), anti-MBP (1:400), anti-S100 (1:400). All slides were evaluated by using an immunofluorescence microscope (Leica).

Histological assessment of typical calcium-dependent signaling on the regenerated nerve. At 12 weeks postoperatively, the regenerated sections of sciatic nerves were used for c-fos and BDNF immunofluorescence assays as described above. The primary antibodies (Abcam, USA) included, anti-fos (1:400) and anti-BDNF (1:1000, Abcam, USA).

Note 9. Functional recovery analysis.

We performed a walking trajectory analysis to measure functional recovery. SFI values were calculated by the formula reported by previous study.[3] SFI ranges from -100 to 0, where -100 indicated complete neurological dysfunction, and 0 indicated good repair. The belly of the gastrocnemius muscle and the proximal nerve were inserted two

electrodes after the regenerated nerve exposing. The CAMP and NCV were calculated based on electromyography.

Supplementary Figures

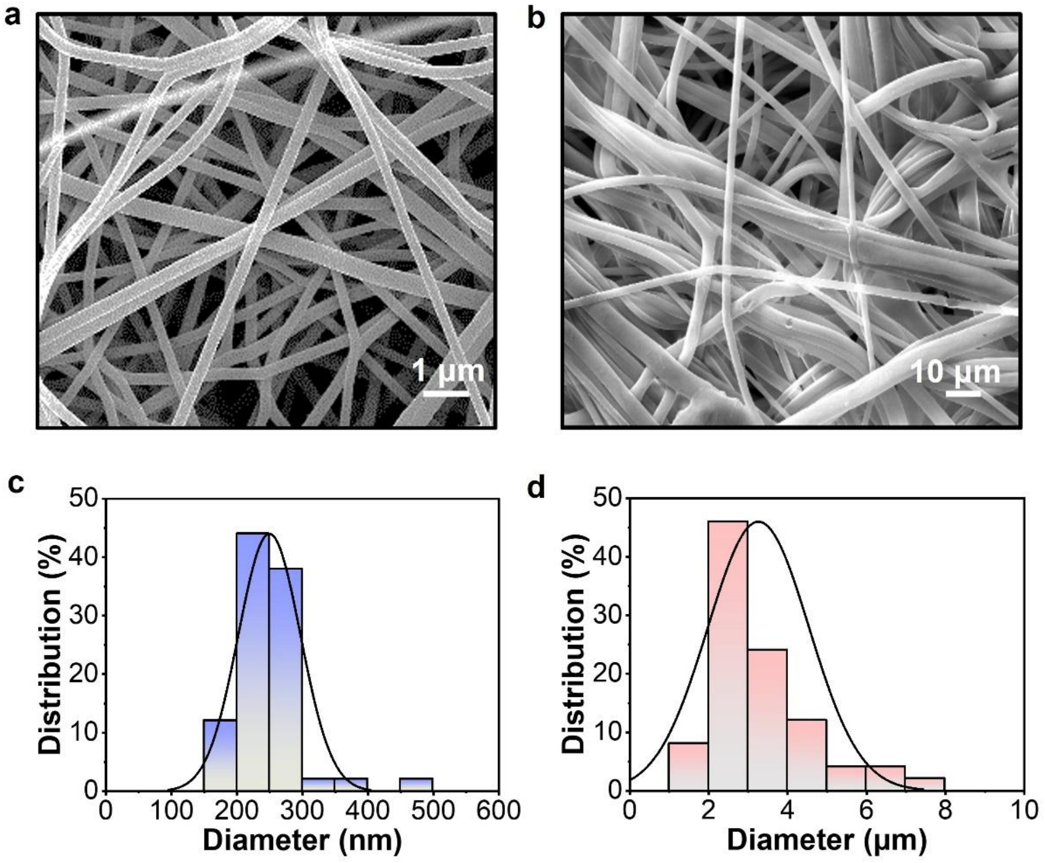

Supplementary Fig. 1 SEM images of (a) PVDF NFs and (b) PP NFs. The fiber diameter statistical distribution of (c) PVDF NFs and (d) PP NFs.

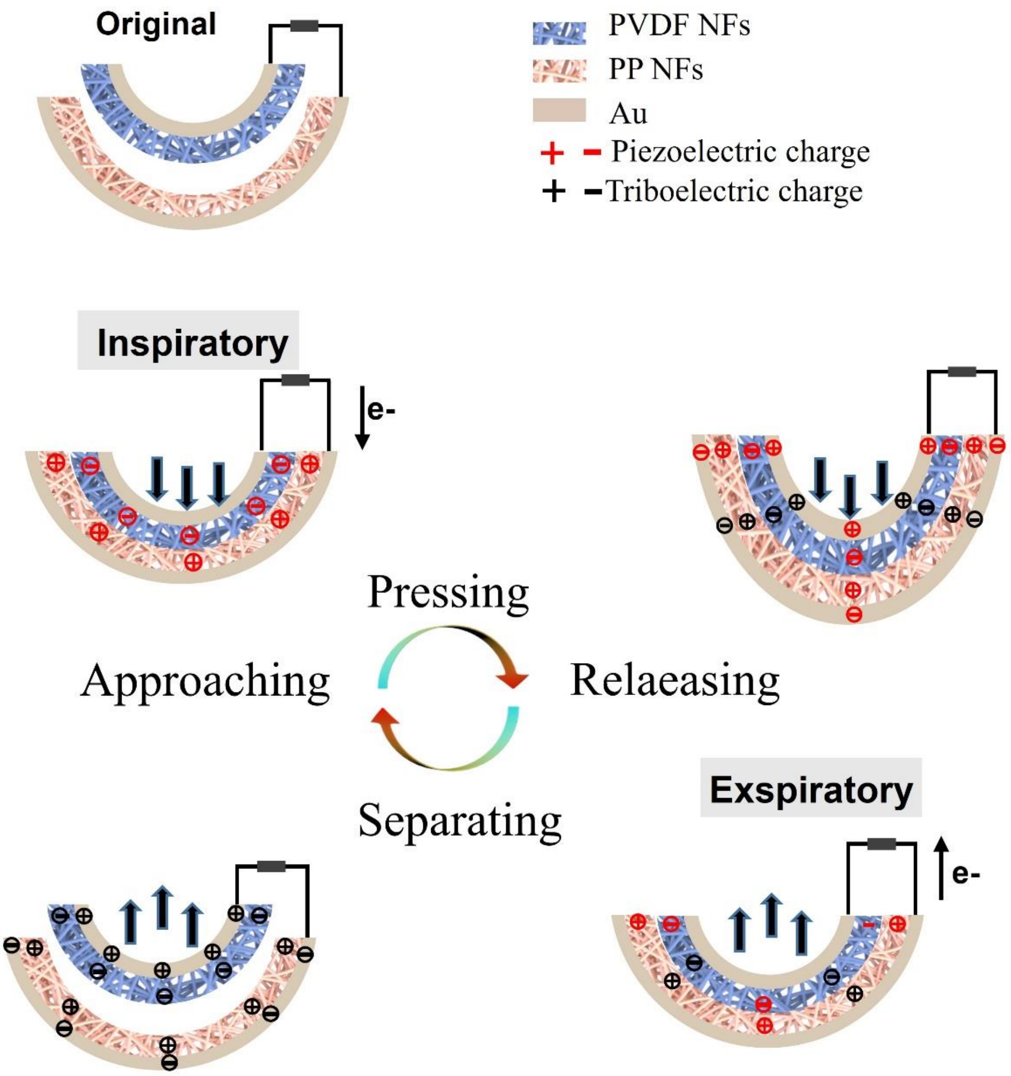

Supplementary Fig. 2 Schematic illustration of the working mechanism of respiratory movement driving the TP-hNG in a complete contact-separation cycle, including inspiratory phase and the exhalation phase.

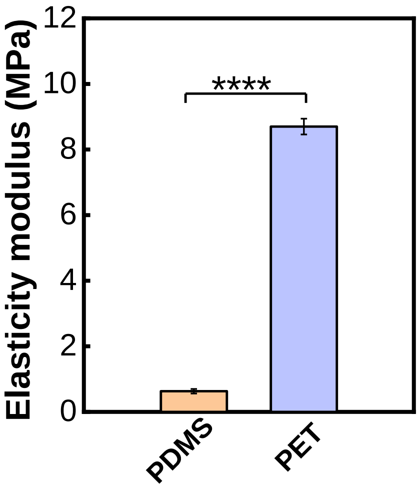

Supplementary Fig. 3 Elastic modulus of outer PET and inner PDMS layers. (n = 5, ****: $p < 0.0001$).

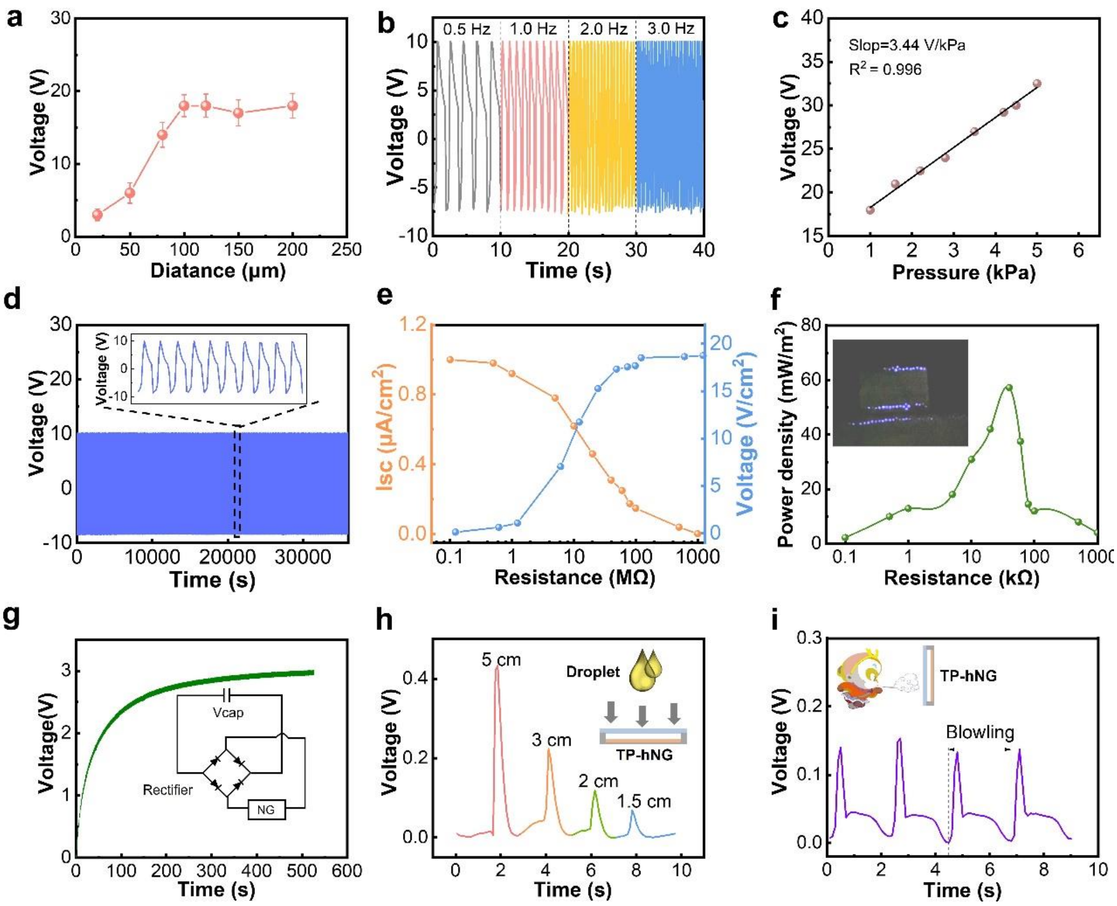

Supplementary Fig. 4 The output performance of TP-hNG. (a) The open-circuit voltage of the TP-hNG under different gap distances at 1 kPa pressure. (b) Open circuit voltage of TP-hNG under the micromechanical force of 0.5-3 Hz and 1 kPa. (c) Linearity relationship between applied pressure and output voltage. (d) Fatigue test via 36000 cycles under 1 kPa pressure. (e) The short current density (Isc) and output voltage of TP-hNG by loading different resistances. (f) The power density of TP-hNG was calculated by connecting different resistance. Inset: 50 LED was lighted by TP-hNG. (g) The capacitor charging curve of TP-hNG under periodic pressure (1 Hz, 1kPa) that was applied by a line motor. Output voltage in response to different ultraweak mechanical deformations caused by water drop (h), human breath (i).

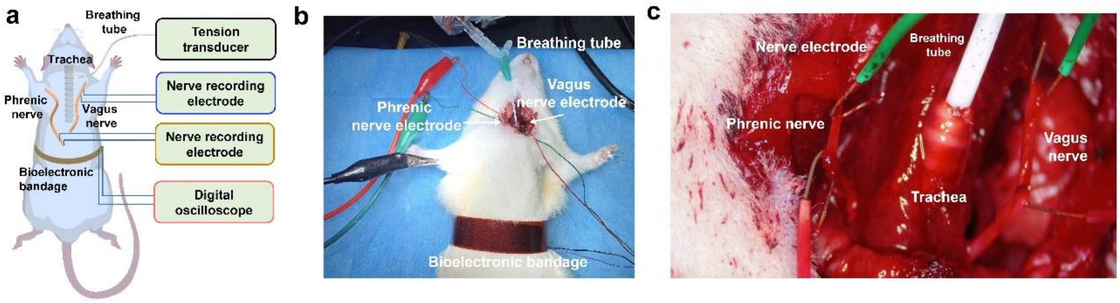

Supplementary Fig. 5 (a) Synchronism measurement between Bio-iES signals and respiratory tract vagus and phrenicus nerve impulses. (b) Surgery image of synchronously measuring tidal volume respiration curve, respiratory tract vagus and phrenicus discharge impulses of adult SD rats. (c) Magnified view of surgery image recording respiratory tract vagal potential and phrenic nerve potential.

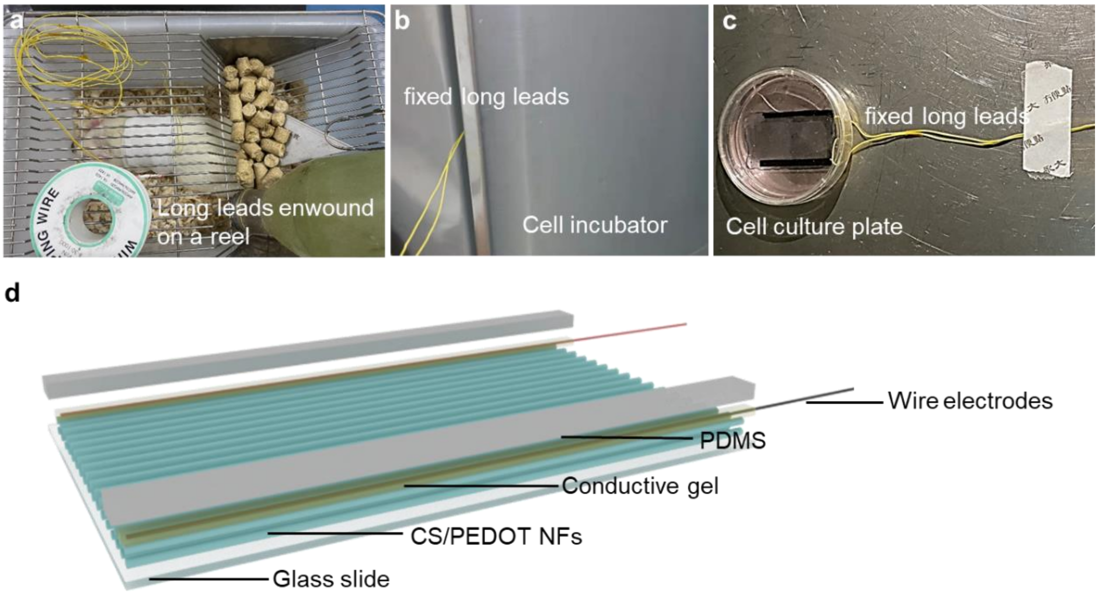

Supplementary Fig. 6 (a) Rats were housed in cages with free access to water and food, and long leads enwound on a reel to allow rats to move freely in daily life. (b) The cell culture dish was placed in a closed incubator. (c) Leads outside of the plate were fixed on the inside of the incubator by sterilized biological tapes. (d) Schematic representation of the artificial device structure in (c) used for the electrical stimulation.

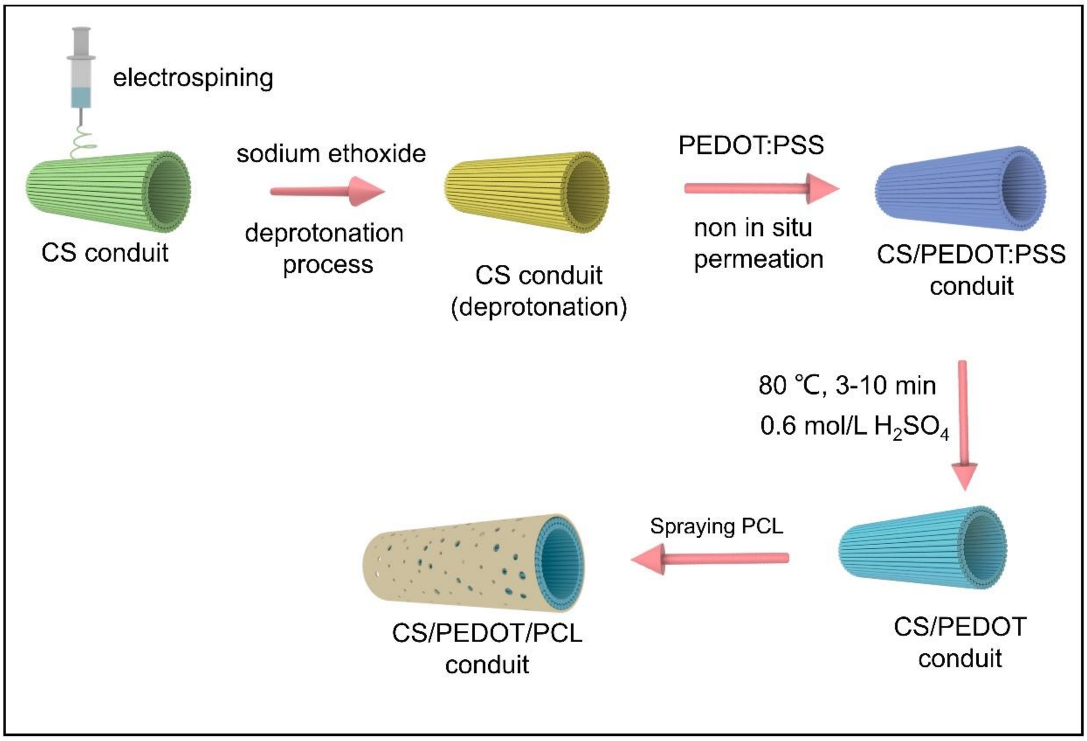

Supplementary Fig. 7 The manufacturing process of the MF-NGC.

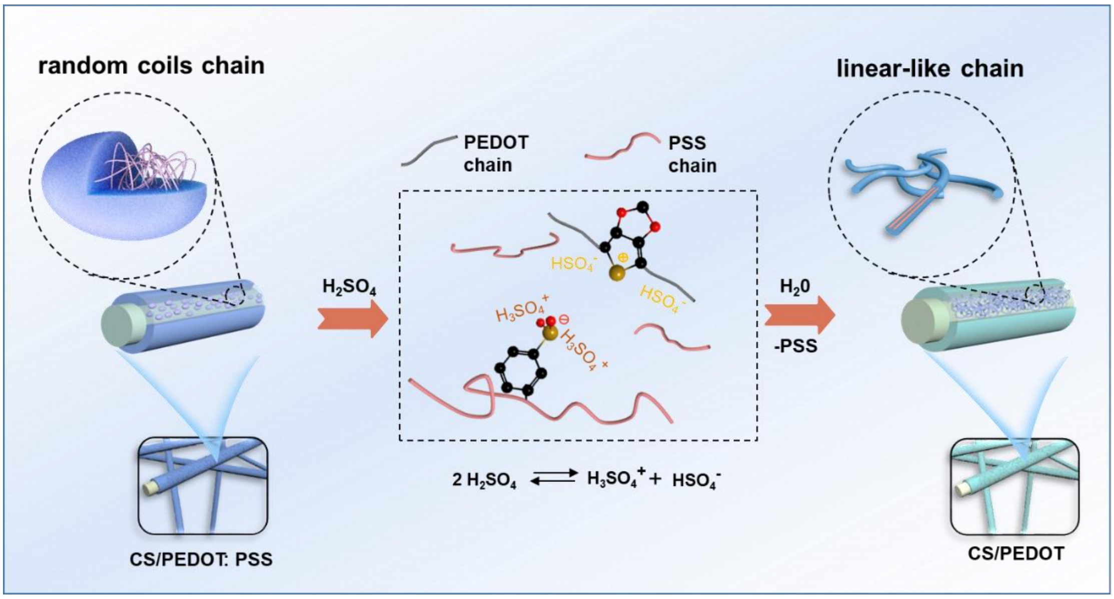

Supplementary Fig. 8 Diagram of the structural rearrangement of PEDOT: PSS. The amorphous PEDOT: PSS grains (left) are reformed into crystalline PEDOT: PSS nanofibrils (right) *via* a charge-separated transition mechanism (middle) *via* a concentrated H_2SO_4 treatment.

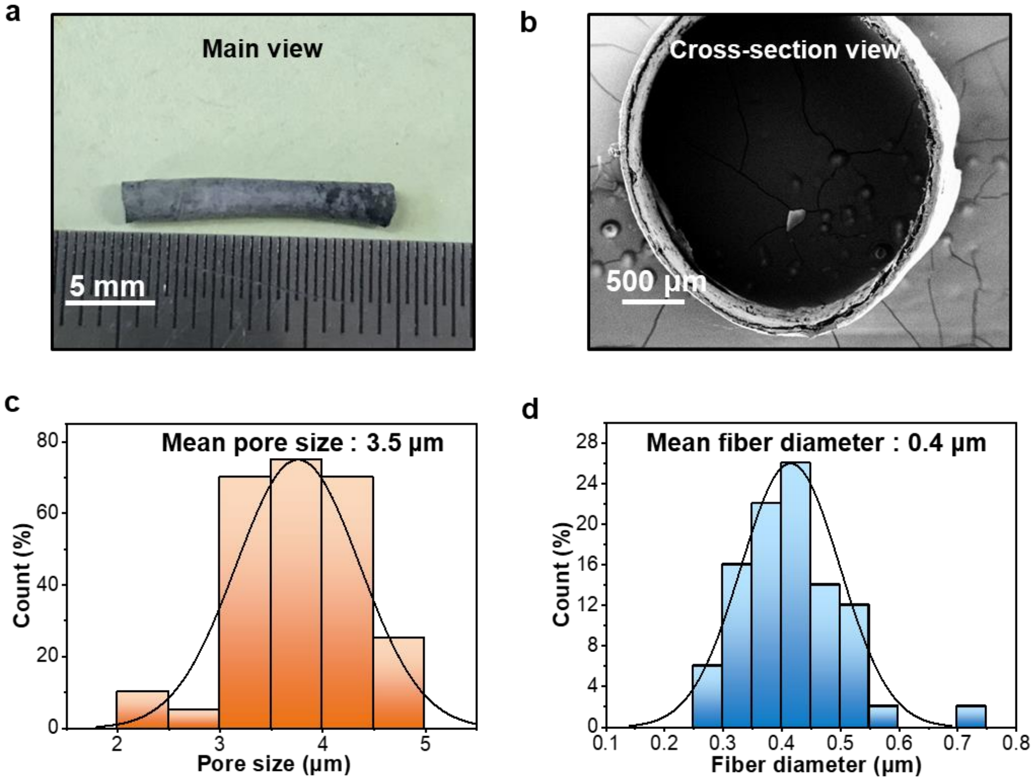

Supplementary Fig. 9 The main view (a) and cross-section view (b) of MF-NGC. (c) The pore size distribution of PCL layers. (d) The fiber diameter distribution of CS/PDOT layers.

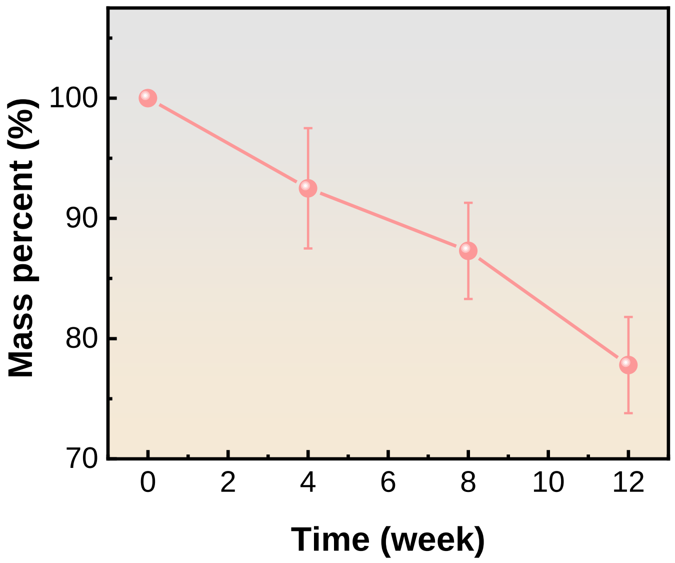

Supplementary Fig. 10 Degradation curve of the MF-NGC.

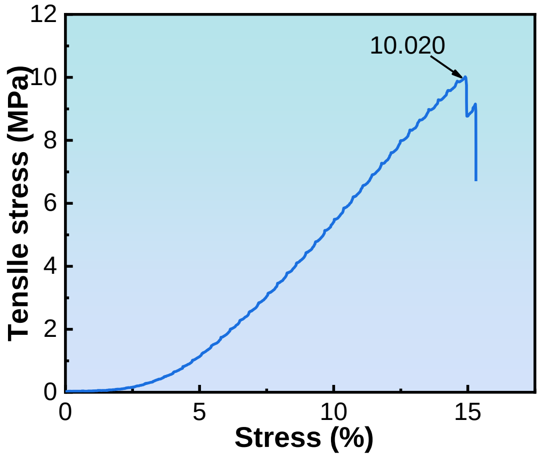

Supplementary Fig. 11 Stress-strain curve of the MF-NGC.

.

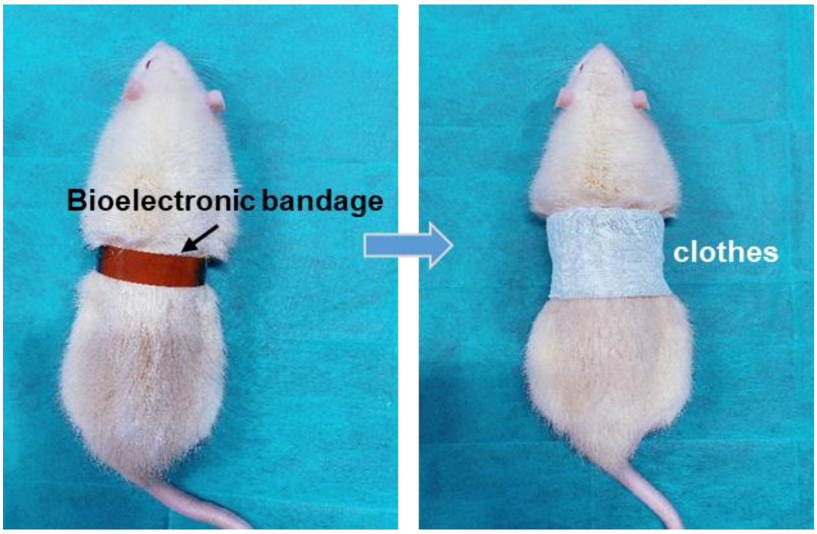

Supplementary Fig. 12 The bioelectronic bandage was fixed on the abdomen of SD rats to be derived by the respiratory movement for accelerating the restoration of PNI. A breathable clothes was worn on the SD rat to avoid slide displacement of bioelectronic bandage during the daily activity.

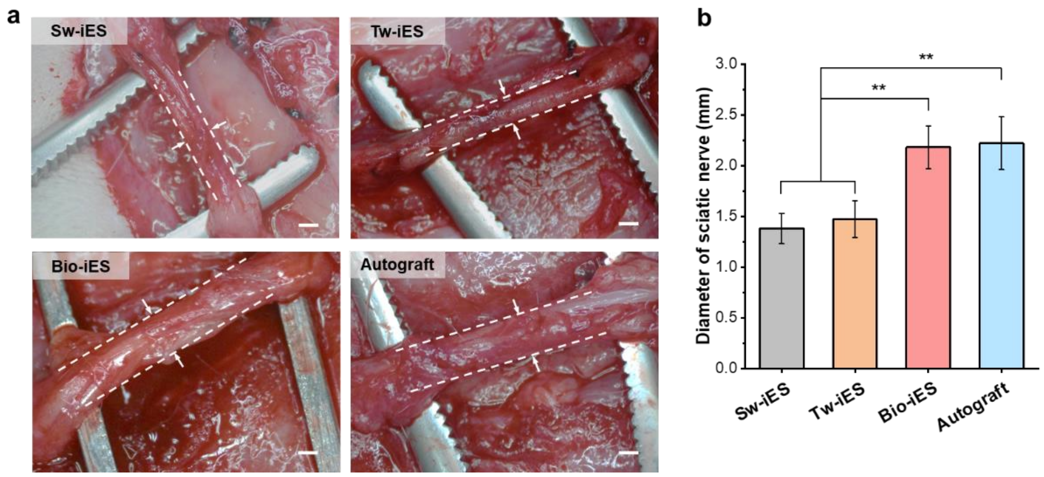

Supplementary Fig. 13 (a) Surgical images of the regenerative nerve in Sw-iES, Tw-iES, Bio-iES, and autograft groups after the postoperative 12 weeks, bar = 1 mm; (b) Diameter of regenerated sciatic nerve calculated from surgical image. (n = 5, **: $p < 0.01$).

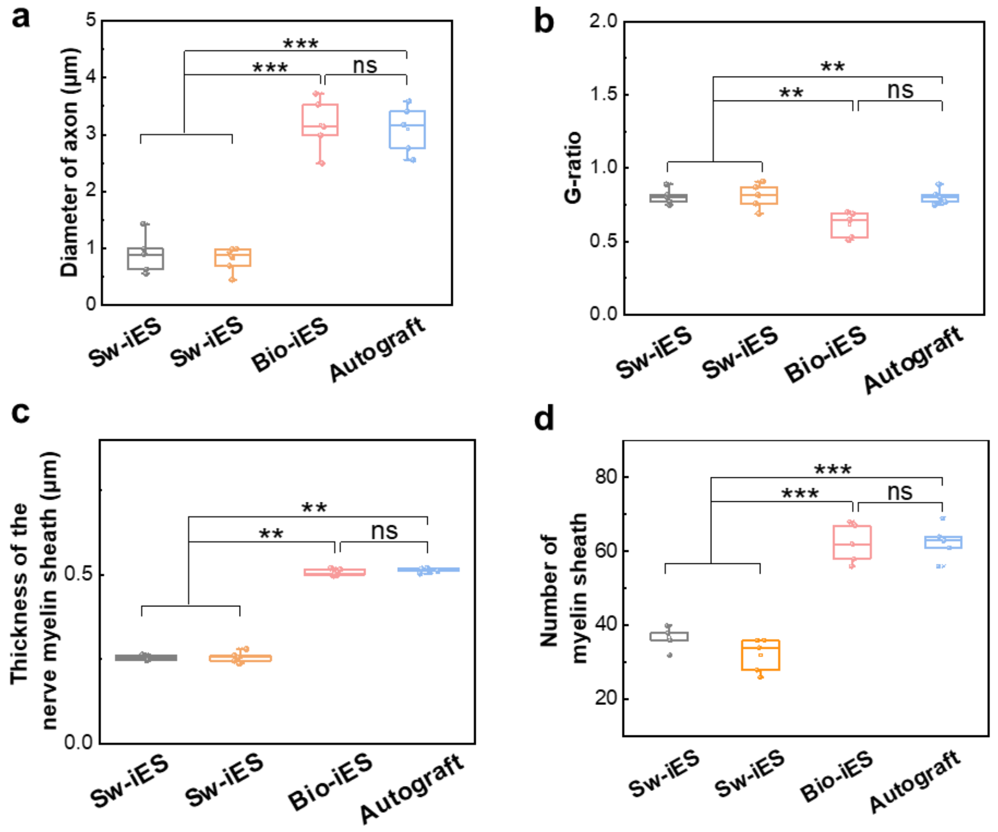

Supplementary Fig. 14 Statistical analysis of regenerated nerve fibers was performed by counting, (a) diameter of axon, (b) degree of myelination (G-ratio), (c) thickness of the nerve myelin sheath and (e) number of myelin sheath layer. Data are expressed as mean values ± S.D. (n = 5, *: $p < 0.05$, **: $p < 0.01$, *** : $p < 0.001$).

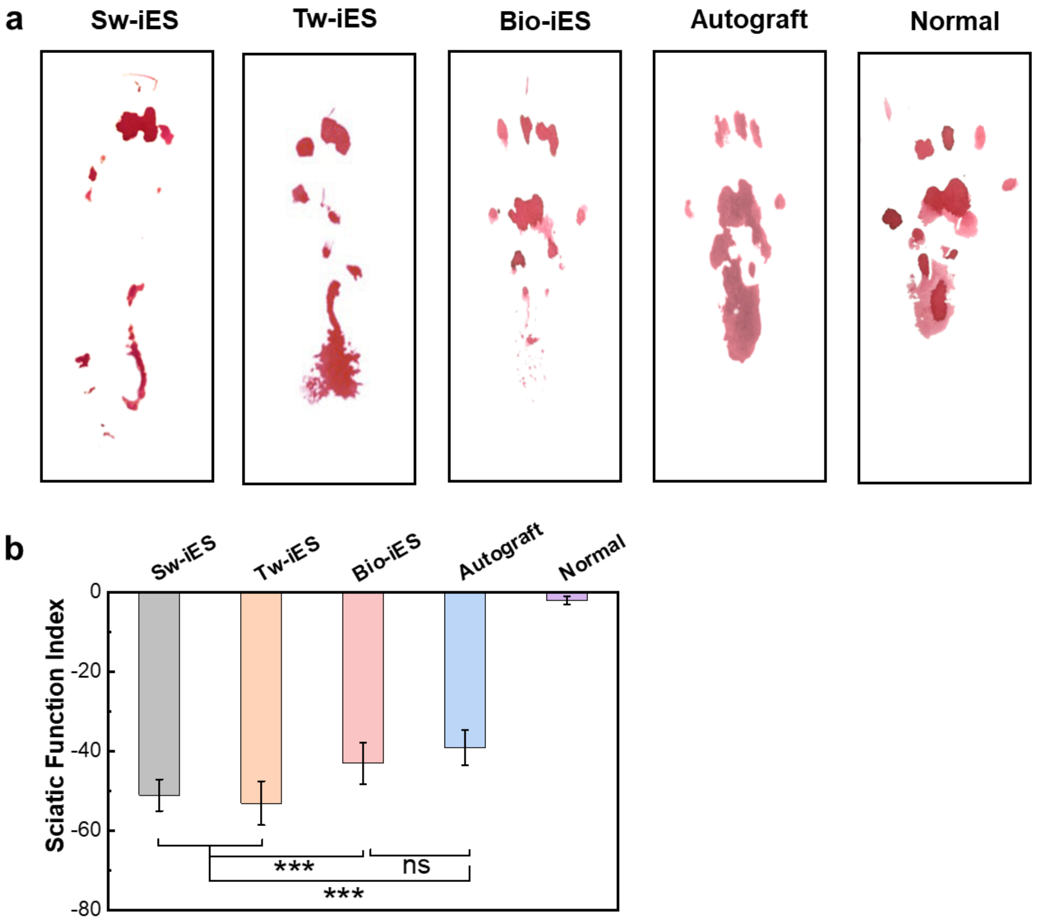

Supplementary Fig. 15 (a) The walking footprint of Sw-iES group, Tw-iES, Bio-iES, autograft, and normal groups at 12 weeks that acquired by the camera. (b) The SFI value was calculated from the walking footprint. ($n = 5$, ***: $p < 0.001$).

Supplementary Tables

Supplementary Table S1. The conductivity comparison before and after removing PSS.

**Materials**	**Conductivity (S/cm)**
CS	0
CS/PEDOT: PSS	0.042
CS/PEDOT	0.37
CS/PEDOT/PCL	0.31

Supplementary Reference

1. Zhu L, *et al.* Aligned PCL Fiber Conduits Immobilized with Nerve Growth Factor Gradients Enhance and Direct Sciatic Nerve Regeneration. *Advanced Functional Materials* **30**, (2020).
2. Du L, *et al.* Design of high conductive and piezoelectric poly (3,4-ethylenedioxythiophene)/chitosan nanofibers for enhancing cellular electrical stimulation. *J Colloid Interface Sci* **559**, 65-75 (2020).
3. Jing W, *et al.* Constructing conductive conduit with conductive fibrous infilling for peripheral nerve regeneration. *Chemical Engineering Journal* **345**, 566-577 (2018).
